# Common Consequences of Uncommon Congenital Heart Disease: Exploring the Trilogy of Fallot With Infective Endocarditis and Cerebral Venous Thrombosis

**DOI:** 10.7759/cureus.45244

**Published:** 2023-09-14

**Authors:** Anshika Mishra, Sonali Singh, Murali M Rama Krishna Reddy, Mohammad Ahsan Anwaar, Anupam S Yadav, Ewuradjoa Ayirebi-Acquah, Ogbonnaya Akuma, Reeju Maharjan, David C Ugwa, Chinaza M Akuma, Nnenna E Ikeogu

**Affiliations:** 1 Pediatrics, King George's Medical University, Lucknow, IND; 2 Pediatric, King George's Medical University, Lucknow, IND; 3 Internal Medicine, Kasturba Medical College Mangalore, Mangalore, IND; 4 Internal Medicine, CMH Lahore Medical College and Institute of Dentistry, Lahore, PAK; 5 Psychiatry, GSVM Medical College, Kanpur, IND; 6 Internal Medicine, Lekma Hospital, Accra, GHA; 7 Internal Medicine, Ebonyi State University, Abakaliki, NGA; 8 Neurology, V.N. Karazin Kharkiv National University, Kharkiv, UKR; 9 Neurology, California Institute of Behavioral Neurosciences and Psychology, Fairfield, USA; 10 Internal Medicine, Richmon Gabriel University, Kingstown, VCT; 11 MPH, College of Health Professions, Chamberlain University, Chicago, USA; 12 Internal Medicine, Abia State University Teaching Hospital, Aba, NGA

**Keywords:** pulmonary stenosis, atrial septal defect (asd), beta-lactam, cerebral venous thrombosis (cvt), trilogy of fallot (tof)

## Abstract

Trilogy of Fallot (ToF) is a rare congenital heart disease characterized by a combination of atrial septal defect, pulmonary stenosis, and right ventricular hypertrophy. It is more common in females and can cause symptoms such as cyanosis and breathlessness. ToF can lead to complications like thromboembolic events, including infective endocarditis (IE) and cerebral venous thrombosis (CVT). This case study discusses a nine-year-old female with ToF who also had IE and CVT. The patient recovered well following treatment with intravenous beta-lactam and aminoglycoside for IE and subcutaneous low-molecular-weight heparin for CVT.

## Introduction

Congenital heart diseases (CHDs) are a group of conditions that affect the heart’s structure and function from birth. Trilogy of Fallot (ToF) is a rare but significant anomaly characterized by three specific heart defects: atrial septal defect (ASD), pulmonary stenosis (PS), and right ventricular hypertrophy (RVH) [1]. ToF presents numerous challenges and can lead to complications that significantly impact a patient’s health. One such case is when ToF is accompanied by two interrelated complications: infectious endocarditis (IE) and cerebral venous thrombosis (CVT). IE involves the infection of the heart’s inner lining, including heart valves. CVT involves blood clots in veins that drain blood from the brain, potentially leading to severe neurological consequences [2,3]. The coexistence of ToF with IE and CVT creates a unique and challenging clinical scenario. This article presents a comprehensive case report that explores the intricate relationship between ToF, IE, and CVT, providing insight into the complex clinical scenario.

## Case presentation

A nine-year-old female with a normal birth history was brought to the pediatric emergency department with a complex medical picture. She had a history of cyanotic spells and breathlessness since childhood, with high-grade fever for the last six days and difficulty breathing. Her breathlessness worsened from New York Heart Association (NYHA) II to NYHA III, and she exhibited a grade-4 systolic murmur with a single S-2. She experienced a focal-onset seizure on the right side of her body, followed by generalized seizures. One day after the seizure, she developed sudden-onset right-sided uncrossed hemiparesis (gradually improving) with motor aphasia.

**Figure 1:**
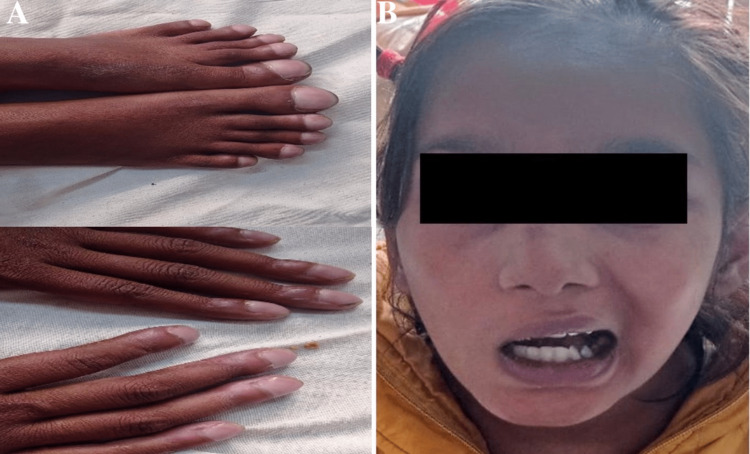
Bilateral hands and feet of grade 3 clubbing (A) and deviation of the angle of the mouth toward the left side, suggesting right upper motor neuron facial nerve palsy (B)

On examination, her sensorium was intact, and no signs of raised intracranial pressure or involvement of other cranial nerves, autonomic function, or bowel and bladder control were noted. She had clubbing of nails of hands and feet characteristic of hypertrophic osteoarthropathy (Figure 1A). Her heart rate was 76/min, pulses were palpable, blood pressure was 110/72 mmHg, respiratory rate was 24/min, and oxygen saturation on room air was 78%, and she had no chest deformities. A neurological system examination revealed reduced power in the right-sided upper and lower limbs, a deviation of the mouth angle toward the left, and sparing of the forehead muscles, suggesting upper motor neuron facial nerve palsy (Figure 1B). The sensory system, cerebellar, and other cranial nerve examinations were normal, and no autonomic system abnormalities were noted.

**Figure 2:**
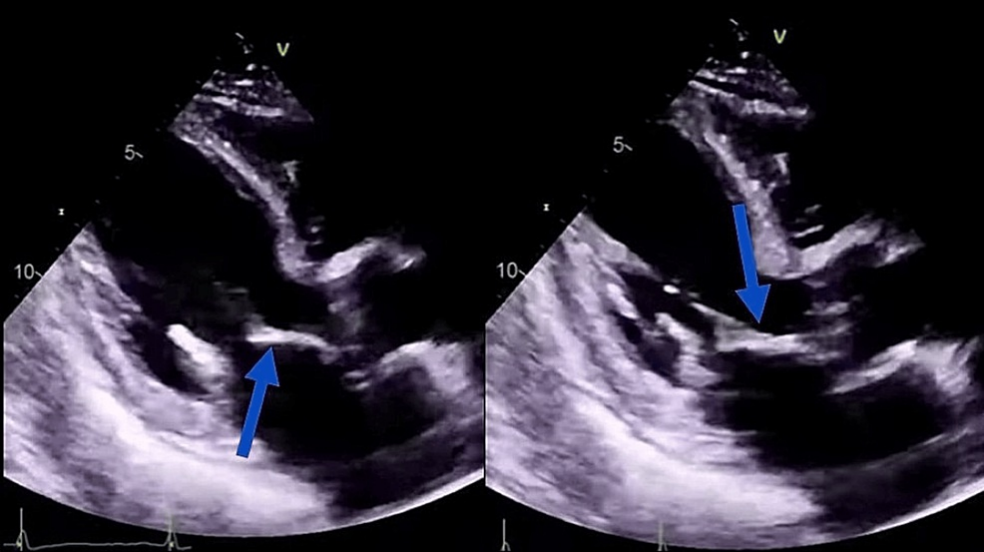
Echocardiogram showing vegetation at tricuspid valve (blue arrow)

**Figure 3:**
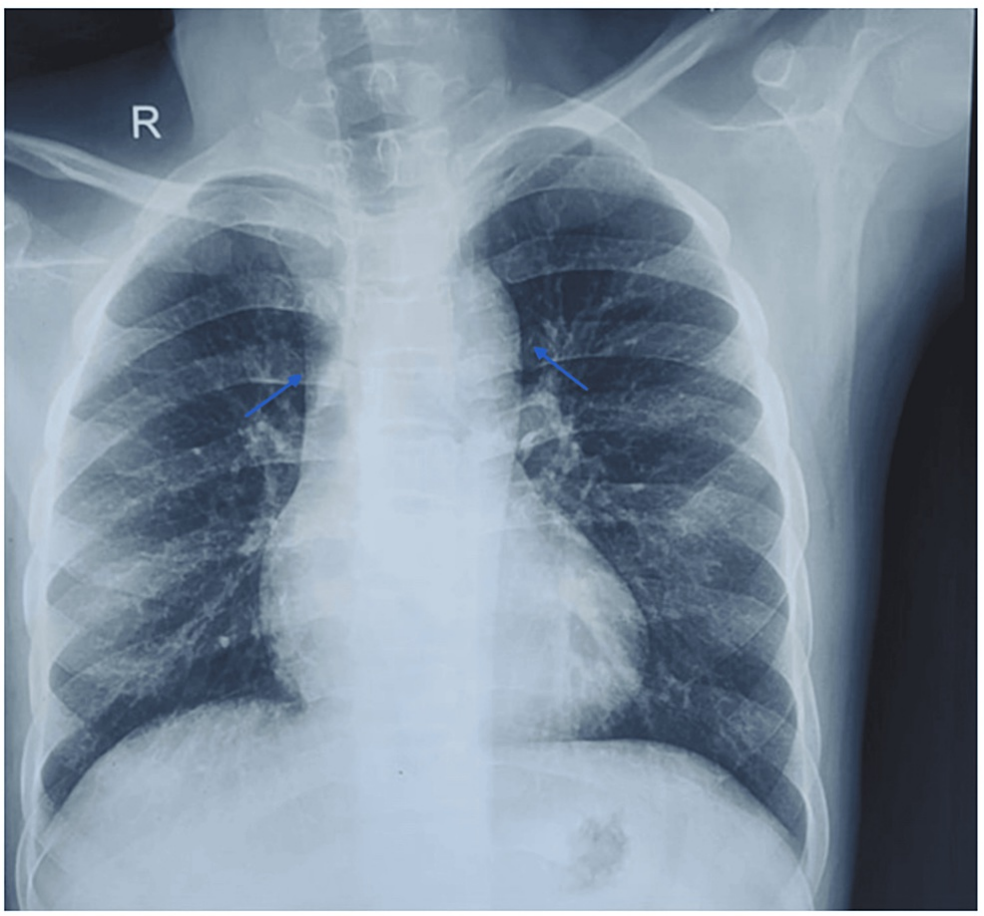
Posteroanterior view of chest radiograph revealing enlarged aorta, concave pulmonary artery, and right ventricular apex (blue arrow)

She had situs solitus, levocardia, and cyanotic CHD characterized by decreased pulmonary flow. Based on the clinical presentation, she was supposed to have tetralogy of Fallot. The electrocardiogram revealed sinus rhythm, and the echocardiogram revealed a large 22-mm ostium secundum, ASD, severe PS, and RVH. Vegetation on the septal leaflet of the tricuspid valve suggested ToF with IE (Figure 2). However, there was no evidence of congestive heart failure. All blood samples testing the presence of bacteremia had been taken by applying a standard technique, three blood cultures divided by an hour had been taken, and she was commenced on empirical intravenous antibiotics. All blood cultures later came out sterile. A chest radiograph revealed an enlarged aorta, concave pulmonary artery, and right ventricular apex (Figure 3).

**Figure 4:**
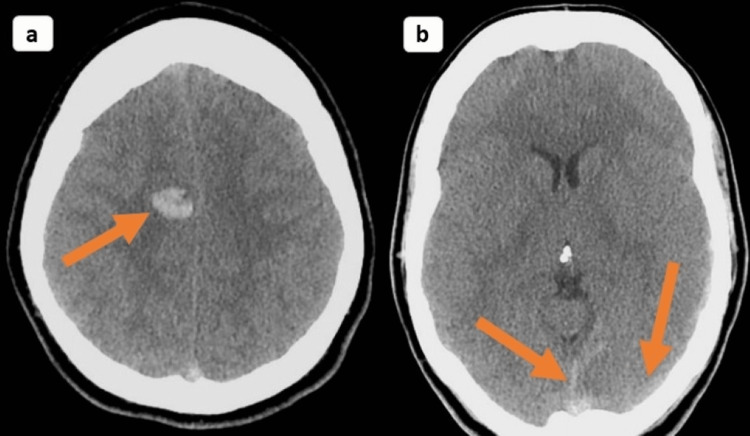
CT revealing a hyperintense signal intensity alteration in the right frontal lobe suggestive of a hemorrhagic infarct and multiple filling defects in the superior sagittal sinus and the transverse sinus suggestive of cerebral venous sinus thrombosis

Computed tomography (CT) of the brain showed hyperintense signal intensity alteration in the right frontal lobe, suggesting a hemorrhagic infarct. Multiple filling defects in the superior sagittal sinus and the transverse sinus suggested cerebral venous sinus thrombosis, as shown in Figure 4.

She was admitted to the intensive care unit and was managed with intravenous fluid, ceftriaxone, and gentamicin. Her condition improved gradually over a 21-day hospital stay, with improvements in right-sided hemiparesis and aphasia in fluency, although facial nerve palsy persisted.

Following her 21-day hospitalization and the notable improvement in her right-sided hemiparesis and aphasia, the patient necessitates a comprehensive long-term care plan. First and foremost, continued anticoagulation therapy with low-molecular-weight heparin (LMWH) is imperative to prevent the recurrence of CVT. The ongoing neurorehabilitation program, encompassing physical, occupational, and speech therapy, is crucial for her journey toward regaining full functionality and language skills. Addressing persistent facial nerve palsy requires specialized care, including consultations with a neurologist and facial nerve specialist, complemented by physical therapy and targeted facial exercises.

Regular follow-up appointments with a multidisciplinary team, comprising neurologists, hematologists (for vigilant anticoagulation management), and therapists, were applied to monitor her progress and make necessary treatment adjustments. Lifestyle modifications, such as adopting a heart-healthy diet, engaging in regular exercise, and managing risk factors like hypertension, were advised for the prevention of future strokes. Moreover, her parents were also counseled for emotional and psychological support, possibly facilitated through counseling or participation in support groups.

## Discussion

Epidemiology

The incidence of ToF ranges from 1.2% to 6% of CHDs diagnosed in children [1,4]. ToF is more commonly seen in females at a ratio of 3:2 [1]. Although the ToF is considered to be an infrequent lesion, growing experience with surgery for CHD has shown it to be more common than previously thought. A study reported that out of the 370 cases of CHD that have come to surgery, 22 were patients with trilogy with an incidence of 6% [1,4]. The age till which a ToF patient may survive is mentioned as around 71 years. Patients as young as seven months of age present with cyanosis [5]. The majority of the patients present within the first year of life with repeated infections, especially respiratory infections, breathlessness, and poor growth. ToF presents a range of clinical features and can vary in severity. The three main components of ToF are ASD, PS, and RVH. ToF is a sporadic CHD reported less often than tetralogy of Fallot in the literature [4]. The interatrial shunting in these cases can be due to patent foramen ovale (PFO), ostium secundum ASD, ostium primum ASD, or sinus venosus type. In contrast, the right ventricular outflow tract obstruction can be due to pulmonary valve stenosis or stenosis of the pulmonary artery and its branches [6]. There are two subclasses of this rare defect. The first subclass is patients with large ASD with mild to moderate PS and left to right shunt. In patients with true ASD, the right ventricular outflow tract obstruction is usually of valvular PS type. The second subclass is those with severe pulmonary valve stenosis and right to left shunt. In these patients, the interatrial shunting from right to left is usually through PFO [7].

Clinical presentation

The patients generally present within the first year after birth with breathlessness, repeated upper respiratory tract infections, syncope, and delayed growth. Increased fatigue, tet spells (sudden bluish discoloration and fainting), and squatting are other symptoms that older children present with. Cyanosis, clubbing, thrill at the apex, a prominent wave in the jugular venous pressure wave, and rarely chest deformity are noted on examination. A systolic murmur at the apex and a diminished or absent pulmonary second heart sound are often heard on auscultation [1]. In ToF, cyanosis develops mainly due to the increasing pressure in the right atrium due to the associated PS, leading to the right-left shunt. This also explains the reason for other symptoms seen in ToF, with patients ultimately developing right ventricular failure [5]. Hypoxia-induced polycythemia increases blood viscosity in patients with cyanotic CHD, leading to increased stasis and septic embolus development [5,6]. The association between erythrocytosis and cerebrovascular events is unclear; complications like IE and CVT due to ToF are uncommon in the literature, unlike in tetralogy of Fallot [8,9]. CVT in tetralogy of Fallot without cerebral artery thrombosis is unusual due to the more prolonged survival of patients with tetralogy of Fallot. Invasive procedures like dental surgeries are the most critical factors associated with the increased risk of IE in CHDs [8]. *Streptococcus* and *Staphylococcus* species are the most common organisms causing IE in CHD patients. IE is suspected in CHD when fever persists for an extended period [2]. Focal neurological deficits in patients with known or recently diagnosed CHD should raise suspicion of CVT, and an unenhanced CT scan of the brain can provide a preliminary diagnosis [3].

Infected vegetation from IE may embolize the bloodstream and cause a thromboembolic event, potentially resulting in a stroke, as in our case [8]. In this case, it is plausible that an embolus from the infected heart valves traveled to the left cerebral cortex, leading to the thromboembolic event and subsequent stroke.

Clinical diagnosis

ToF is diagnosed through transthoracic 2D echocardiography, which may show evidence of ASD, PS, and RVH. IE focus is also observed, and a blood culture confirms the organism. Electrocardiography may demonstrate RVH with normal sinus rhythm or atrioventricular (AV) block [2]. Right atrial enlargement may also occur with flow reversal across ASD. An electrocardiogram may show an enlarged right atrium with a right axis deviation. Chest X-ray will reveal oligemic lung fields and a prominent pulmonary trunk. An echocardiogram confirms PV stenosis and a stretched PFO. However, in cases of large ASD with mild to moderate PS, the electrocardiogram will show RV hypertrophy; the chest X-ray will show increased pulmonary arterial blood flow, a dilated pulmonary trunk, and a dilated right ventricle [5]. Chest radiographs may reveal right atrial enlargement, a prominent pulmonary artery, and a clear lung field in the periphery.

Treatment recommendations

Fallot’s trilogy can be managed by combining treatment and surgical management. Medical management involves parents’ education as well as drug therapy. Tet spells can be managed by placing the infant in a knee-to-chest position to improve blood flow to the lungs. Depending on the patient’s clinical condition, medical therapy includes prostaglandin E1 diuretics and supplemental oxygen [8]. It’s important to note that the specific treatment plan and timing can vary based on the individual patient’s condition and the recommendations of their medical team.

Cardiac catheterization plays an essential role in the management of patients with ToF, as well as the tetralogy of Fallot. Several palliative transcatheter interventions can be performed in the neonatal period to allow for improved oxygen saturations and interval growth of the pulmonary arteries until corrective surgery is performed. Most patients develop branch pulmonary artery stenosis, right ventricular outflow tract obstruction, pulmonary insufficiency, or significant residual left-to-right shunts during long-term follow-up after corrective surgery. Transcatheter interventions can be performed to treat many of these issues, often eliminating or delaying the need for subsequent surgery. The indications for cardiac catheterization and the specifics for various interventional procedures for patients depending on specific treatment plans and timing can vary based on the individual patient’s condition and the recommendations of their medical team [10].

Surgical management is complete repair of the underlying defect, which may include closing the ASD with a patch and enlarging the pulmonary valve or the passage to the pulmonary artery. A temporary shunt may be placed to improve blood flow to the lungs before the complete repair. Follow-up care is obligatory to monitor for complications, such as residual defects, arrhythmias, or pulmonary valve problems [11]. Surgery remains the mainstay in treating ToF, repairing ASD with closure, and repairing PS with valvuloplasty [1]. According to recent guidelines, antibiotic prophylaxis for IE is not recommended for CHDs and recommends oral hygiene. Amoxicillin is the first-line prophylaxis for IE at 50 mg per kg for children and 2 g for adults daily. Azithromycin, clarithromycin, cefazolin, and ceftriaxone are the alternatives. A combination of beta-lactams and aminoglycosides for at least two weeks is the recommended first-line therapy for IE [2]. In our patients, we saw excellent responses with ceftriaxone and gentamicin. CVT in children is generally treated with either parenteral unfractionated heparin, subcutaneous low LMWH, or oral warfarin, followed by long-term anticoagulation with LMWH or warfarin for three to six months [3,10]. Our patient tolerated LMWH well for CVT.

## Conclusions

ToF is a rare congenital heart defect characterized by ASD, RVH, and PS, which can lead to chronic hypoxia and erythrocytosis, which increases blood viscosity and the risk of septic embolus. Fever and signs of sepsis may indicate IE, while focal neurological deficits may suggest CVT in patients with known or recently diagnosed CHD. Prompt recognition and appropriate management of IE and CVT are crucial in patients with CHD to prevent complications and improve outcomes.
